# Metagenomic mining reveals novel Cas12 subtypes and their evolutionary diversification

**DOI:** 10.1002/imo2.70057

**Published:** 2025-10-07

**Authors:** Meixia Yu, Zilong Zhao, Peiyang Li, Yunpeng Teng, Feng Xu, Zhimeng Li, Zhijia Yang, Hainan Zhao, Huanbin Zhou, Lanqin Xia, Jinsheng Lai, Beibei Xin

**Affiliations:** ^1^ State Key Laboratory of Maize Bio‐breeding, National Maize Improvement Center, Department of Plant Genetics and Breeding China Agricultural University Beijing China; ^2^ Frontiers Science Center for Molecular Design Breeding (Ministry of Education) China Agricultural University Beijing China; ^3^ State Key Laboratory for Biology of Plant Diseases and Insect Pests, Institute of Plant Protection Chinese Academy of Agricultural Sciences Beijing China; ^4^ Ministry of Agriculture and Rural Affairs Key Laboratory of Gene Editing Technologies (Hainan), National Nanfan Research Institute Chinese Academy of Agricultural Sciences Sanya China; ^5^ Scientific Observing and Experimental Station of Crop Pests in Guilin Ministry of Agriculture and Rural Affairs Guilin China; ^6^ State Key Laboratory of Crop Gene Resources and Breeding, Institute of Crop Sciences (ICS) Chinese Academy of Agricultural Sciences (CAAS) Beijing China; ^7^ Center for Crop Functional Genomics and Molecular Breeding China Agricultural University Beijing China; ^8^ Sanya Institute of China Agricultural University Sanya China; ^9^ Ministry of Agriculture and Rural Affairs Key Laboratory of Gene Editing Technologies (Beijing) Beijing China

## Abstract

We identified 4112 Cas12 Proteins and 6 new Cas12 subtypes, revealed their significant diversity in N‐terminal regions, repeat sequences, and sequences motifs. We developed an AI‐driven algorithm, Cas12fam, which allows precise annotation of 18 distinct domains in Cas12 proteins. Through detailed structural analysis, we identified two novel glycine residues in the RuvC‐like nuclease domains (RuvC‐I, RuvC‐II), which were experimentally validated to play a critical role in the DNA‐cleavage activity of Cas12. Our analyses revealed that Cas12 proteins are enriched in specific environments, with significant variation in their abundance across ecological niches.

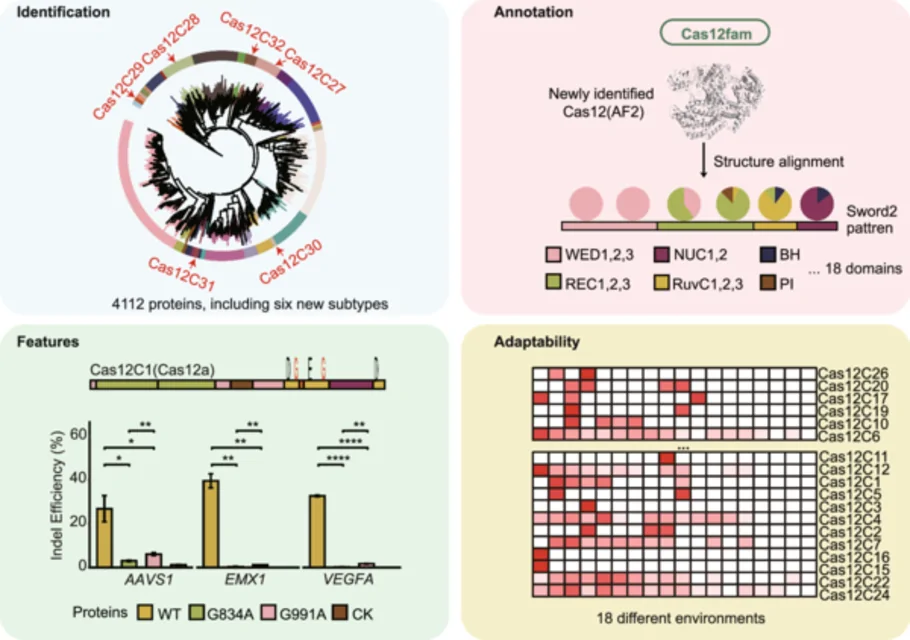

To the editor,

The clustered regularly interspaced short palindromic repeats (CRISPR) and CRISPR‐associated (Cas) system, a key outcome of host‐virus co‐evolutionary dynamics, is widely distributed across prokaryotes [1]. CRISPR/Cas systems are divided into two classes, six types, and 33 subtypes based on their effector modules [2]. Class II systems, although less abundant than Class I, employ a single multi‐domain effector protein, making them more streamlined for genetic engineering [3, 4]. Among these, type V CRISPR‐Cas systems are notable for their diversity, encompassing 15 subtypes [5]. Cas12 nucleases in type V systems exhibit remarkable variability, utilizing distinct CRISPR RNA (crRNA) maturation mechanisms, including self‐processing, trans‐activating crRNA (tracrRNA)‐dependent and short‐complementarity untranslated RNA (scoutRNA)‐dependent, respectively. They target diverse substrates for editing, including double‐stranded DNA, single‐stranded DNA, and RNA, respectively. Some Cas12 subtypes perform specialized functions such as transposon‐guided DNA integration and nuclease‐independent transcriptional repression [6]. Additionally, Cas12 subtypes occupy varied ecological niches, from groundwater to polluted environments [7, 8].

Despite the growing understanding of type V systems, two primary challenges persist. First, precise prediction of novel type V systems is essential for advancing experimental research. Previous studies focused on effector module architectures, annotating limited domains (e.g., RuvC) using Pfam database searches [9]. Second, although the expansion of genomic and metagenomic datasets has enriched our understanding of type V systems [2], a complete catalog of their ecological distribution remains elusive.

In this study, we analyzed 8 terabytes (TB) of bacterial and archaeal genomic as well as metagenomic data to identify 4112 Cas12 orthologs. We then developed the Cas12 Family Annotation Method (Cas12fam), for functional domain annotation of Cas12 variants, and validated two key conserved glycine residues in the RuvC‐I and RuvC‐II domains. Moreover, we proposed a scalable nomenclature system for Cas12 subtypes while exploring their evolutionary patterns and ecological distribution, establishing a basis for future research.

## 1 Refining nomenclature for and exploring diversity in type V systems

To explore the diversity of the Cas12 family, we conducted a metagenomic analysis using 8 TB data from The Integrated Microbial Genomes & Microbiomes (IMG/M: https://img.jgi.doe.gov/m/) and the National Center for Biotechnology Information (NCBI) GenBank (https://www.ncbi.nlm.nih.gov/genbank/). A bioinformatics pipeline annotated over 3.1 billion proteins, identifying ~6 million near CRISPR arrays (Figure S1A). PSI‐BLAST detected 4112 Cas12 orthologs (Table S1), uncovering 6 novel type V systems which have not been documented yet (Table S2 and Figure 1A). Traditional Cas12 nomenclature, appending letters for the same subtypes [10], is limited by the alphabet. We propose the Cas12Cn system, where “C” denotes the Cas12 cluster and “n” an Arabic numeral, providing a scalable framework for the growing diversity of Cas12 effectors.

**FIGURE 1:**
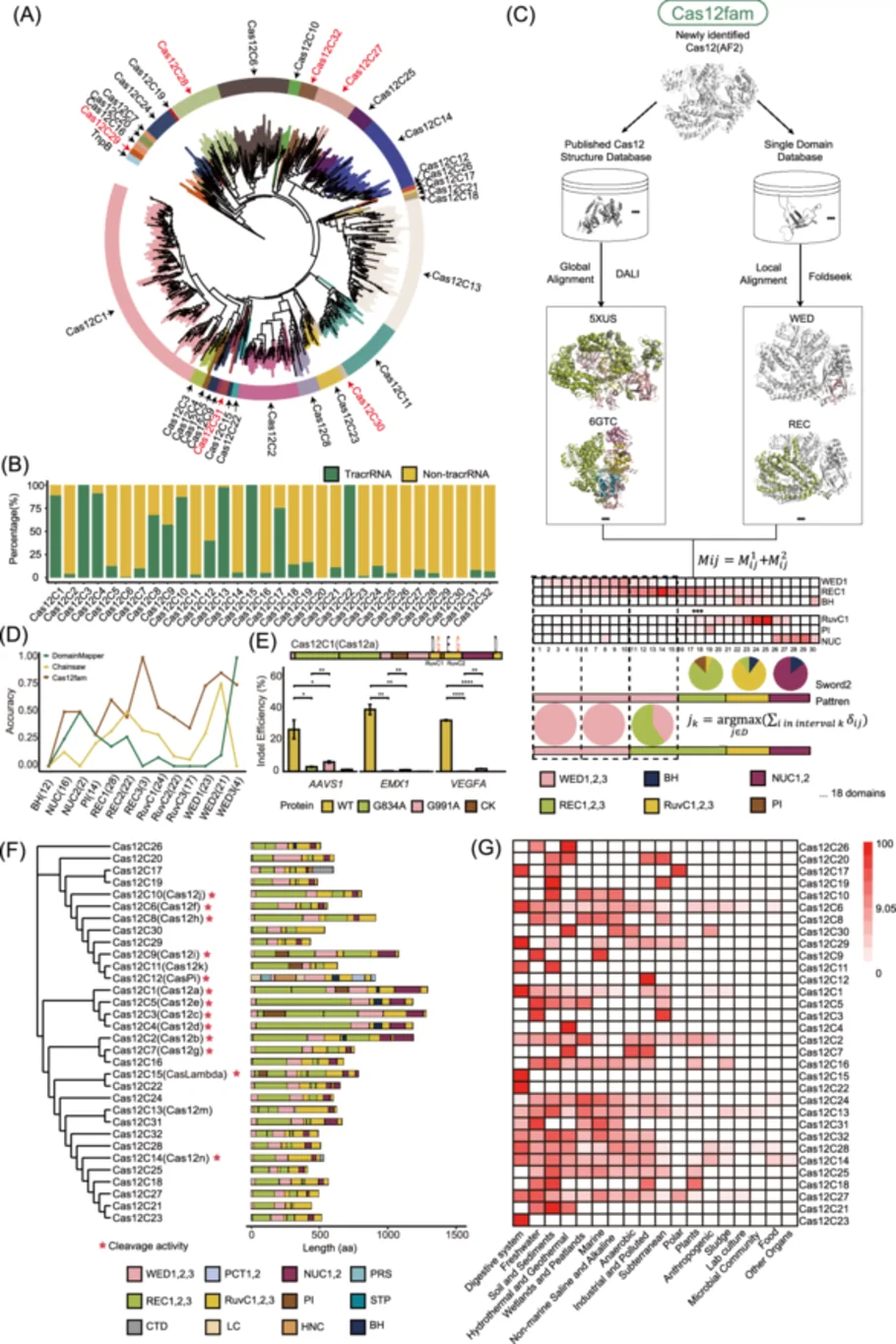
Phylogenetic, structural, and ecological profiling of Cas12 subtypes. (A) Phylogenetic tree of Cas12 effector. Branches are color‐coded to indicate different Cas12 subtypes. Newly‐identified subtypes are labeled as Cas12C27 through Cas12C32 and highlighted in red. (B) Predicted dependence on tracrRNA across Cas12 subtypes. Stacked bars show the percentage of loci encoding a canonical tracrRNA (yellow) versus those predicted to be tracrRNA‐independent (green). (C) The workflow for annotating newly identified Cas12 proteins using the Cas12fam approach. (D) A comparison of domain annotation performance across three tools: DomainMapper (green), Chainsaw (yellow), and Cas12fam (brown). The *x*‐axis lists domains (with numbers in parentheses indicating the count of domains evaluated). (E) Indel efficiency of Cas12C1 mutants. Key conserved motifs in Cas12C1 were highlighted in the schematic figure above. The wild type (WT) protein refers to the unmutated Cas12a, while CK (negative control) represents a background condition. Data are presented as the mean ± standard deviation (s.d.) from *n* = 3 biological replicates with significance determined by a two‐tailed Student's *t*‐test (*p < 0.05, **p < 0.01, ****p < 0.0001). (F) Phylogenetic relationships and domain organization of Cas12 subtypes. For each subtype, the colored bar depicts its domain architecture and total protein length (amino acids, aa). Subtypes marked with red stars indicate cleavage activity. (G) The heatmap showing the relative abundance and distribution of various Cas12 subtypes across different environments. The color intensity represents the relative abundance, with darker shades indicating higher concentrations of specific Cas12 subtypes within particular habitats. In this figure, several domain abbreviations are used to represent key structural components of Cas12 proteins. These include: Wedge domain (WED), Bridge domain (BH), Nuclease domains (NUC), Recognition domain (REC), RuvC‐like nuclease domain (RuvC), PAM interacting domain (PI), Casπ C‐terminal domain (PCT), Proline‐rich string domain (PRS), Stop domain (STP), C‐terminal domain (CTD), Lock‐catch domain (LC), a chimera of Helical‐I and NTSB domains (HNC).

The Cas12 family exhibits an average sequence identity of 30.54% across 32 families—lower than 33.3% in Cas9 families (Figure S1B). Cas12 subtypes vary in protein sizes, from Cas12C6 (300–700 aa) to Cas12C2 (1100–1500 aa). The length of C‐terminal lobes for DNA processing is conserved, while N‐terminal lobes involved in recognition vary, likely due to evolutionary insertions reducing RNA dependence (Figure S1C) [8]. Subtypes also differ in repeat lengths and GC contents. The 3' terminal regions of Cas12 repeats frequently feature an A[C/G] motif. Subtypes such as Cas12C4 (Cas12d), Cas12C5 (Cas12e), Cas12C11 (Cas12k), and Cas12C12 (Casπ), which rely on tracrRNA, exhibit a conserved AAAC/AAAG motif. Notably, this motif was also identified in the repeats of additional subtypes, which are predicted to rely on tracrRNA based on in silico analysis (Figure 1B, Figures S1D, and S2).

These diverse characteristics collectively facilitate the classification of Cas12 subtypes. Traditional sequence similarity methods are limited, distinguishing only a subset of these subtypes. Specifically, t‐distributed Stochastic Neighbor Embedding (t‐SNE) analysis based on sequence and structural similarities successfully distinguished 22 out of 32 Cas12 subtypes. The remaining 10 subtypes can be differentiated by additional features of protein families, such as repeat motifs, sequence motifs, and protein size. For example, subtypes like Cas12d (Cas12C4) and Cas12f (Cas12C6) can be more effectively separated by their conserved and unique repeat sequences (Figure S1E,F).

## 2 Artificial intelligence (AI)‐driven annotation of the functional domains of cas12 proteins

We predicted AlphaFold2 structures for 4112 Cas12 proteins, with 4035 achieving predicted local distance difference test scores ranging from 70.02 to 96.32. Cas12 proteins exhibit a bilobed architecture: the recognition (REC) lobe (REC1 and REC2 domains) handles DNA binding and recognition, while the nuclease (NUC) lobe (wedge (WED), RuvC, and NUC domains) drives catalysis and cleavage [11].

We developed Cas12fam, a structure‐based tool that annotates 18 domains in Cas12 proteins. It performs global and local alignments of AlphaFold2‐predicted Cas12 structures against published Cas12 structures (Table S3) and refines NUC, RuvC, and WED boundaries via sliding window (Figure 1C). Leave‐one‐out validation on 21 published structures demonstrated up to 91% accuracy per protein (annotated domains relative to total), with an average of 51% (Table S3). Cas12fam outperformed other AI‐based annotators like DomainMapper [12] and Chainsaw [13] in key domains (Figure 1D), particularly WED (78% accuracy), attributable to its conserved barrel‐like oligosaccharide‐binding domain (Figure S3) [14].

Analyzing 4112 proteins revealed conserved motifs beyond the major DED triad: a DXG motif in RuvC‐I, featuring a glycine adjacent to the active‐site aspartate (as in Cas9 [15]) and another glycine in RuvC‐II, distinctive to Cas12. Notably, these motifs are present in most subtypes except Cas12C2, C8, C15, and C22 (Figure S4). Mutations at these glycines reduced Cas12a cleavage activity (Figure 1E). Cas12fam excels in conserved domains but falters on short/variable regions and depends on prediction quality. These limitations suggest avenues for future enhancements.

## 3 Ecological and evolutionary dynamics of cas12 subtypes

The 4112 Cas12 proteins span 18 environments, showing habitat‐specific variations. Digestive system accounted for 43.80% of Cas12 proteins, freshwater for 9.88%, and soil and sediment samples for 9.31%. Cas12 proteins were significantly enriched in digestive systems, industrial and polluted, anaerobic, hydrothermal and geothermal, non‐marine saline and alkaline, and subterranean environments (Figure S5A). Saturation analysis via random metagenome sampling revealed plateauing discovery curves in wetlands and peatlands, food and other organs environment, suggesting that Cas12 diversity is largely saturated in these niches. Sludge reaches 95% saturation quickly, implying low diversity. Digestive and anaerobic environments show ongoing rises, marking them as reservoirs for the yet‑unexploited Cas12 variants, enriched relative to metagenomic backgrounds (Figure S5B). Several subtypes, including Cas12C1 (Cas12a), Cas12C2 (Cas12b), Cas12C6 (Cas12f), Cas12C13 (Cas12m), Cas12C14 (Cas12n), Cas12C16, Cas12C24, Cas12C25, Cas12C27, Cas12C28, and Cas12C32, were identified across diverse environments, indicating broad ecological adaptability (Figure 1F,G). Our analysis relies mainly on public metagenomic datasets and assembled genomes, which are inherently biased towards certain environments (e.g., human/animal‐associated habitats) and likely underestimate Cas12 diversity in under‐sampled extreme environments like polar regions.

Phylogenetic analysis of 32 subtypes, incorporating Cas12fam domains, aligns with prior findings [2, 16], showing affinities such as between Cas12C1 (Cas12a) and Cas12C5 (Cas12e), and between Cas12C3 (Cas12c) and Cas12C4 (Cas12d). Cas12C15 and Cas12C22 formed a clade enriched in rumen samples, suggesting shared ancestry. Selection pressures also varied with environment. Cas12C12, which carries “lock‐catch” (LC)/proline‐rich string (PRS)/chimera (HNC) insertions, showed Ka/Ks > 1 in polluted environments [8], consistent with adaptive evolution, whereas Cas12C3 exhibited low Ka/Ks (purifying selection) in benign freshwater/groundwater. These patterns illustrate how environments shape Cas12 evolution, fostering adaptations for niche‐specific challenges (Figure 1G and Figure S5C).

By mining 8 TB of genomic and metagenomic data, we assembled a catalog of 4112 Cas12 orthologs, proposed a scalable subtype nomenclature (Cas12Cn), and introduced Cas12fam—an AI‐driven, structure‐based annotator that delineates 18 domains and outperforms other AI‐based domain annotation tools. Integrating these annotations with comparative analyses revealed two conserved glycines in RuvC‐I and RuvC‐II; mutagenesis of Cas12a reduced DNA‐cleavage activity, supporting their functional relevance. Ecological profiling across 18 environments showed strong habitat stratification—with enrichment in digestive, anaerobic, and polluted settings—and rarefaction indicated near‐saturation in some niches but continued discovery potential in others. Phylogenetics analysis recapitulated known relationships and, together with environment‐linked Ka/Ks patterns, underscored how ecological context shapes Cas12 evolution. Collectively, these results provide a unified framework connecting domain architecture, evolutionary, and environmental distribution, offering practical guidance for discovering Cas12 effectors for genome engineering.

## AUTHOR CONTRIBUTIONS

**Meixia Yu**: Investigation; conceptualization, writing original draft; visualization; software; project administration. **Zilong Zhao**: Investigation; conceptualization, writing original draft; visualization; data curation. **Peiyang Li**: Investigation. **Yunpeng Teng**: Investigation; writing original draft. **Feng Xu**: Software; visualization. **Zhimeng Li**: Data curation. **Zhijia Yang**: Writing—review and editing; supervision. **Hainan Zhao**: Writing—review and editing; supervision. **Huanbin Zhou**: Writing—review and editing; **Lanqin Xia:** Writing—review and editing; **Jinsheng Lai**: Conceptualization, writing—review and editing; supervision. **Beibei Xin**: Conceptualization (lead); funding acquisition; writing—review and editing; visualization; project administration; resources; supervision. All authors have read the final manuscript and approved it for publication.

## CONFLICT OF INTEREST STATEMENT

The authors declare no conflicts of interest.

## ETHICS STATEMENT

No animals or humans were involved in this study.

## Supporting information


**Figure S1.** Identification and characterization of Cas12 effectors from metagenomic data.
**Figure S2.** Sequence logos of direct repeat in Cas12 subtypes.
**Figure S3.** Structural overview and comparative analysis of the REC lobe across Cas12 subtypes.
**Figure S4.** Functional motifs identified in Cas12 subtypes.
**Figure S5.** Distribution, diversity, and selection pressure analysis of Cas12 proteins.

**Table S1.** Protein sequences and information for representatives of 32 Cas12 subtypes.
**Table S2.** The nomenclature and counts of type V effectors.
**Table S3.** Annotation results for published Cas12 proteins using the leave‐one‐out strategy.
**Table S4.** Tagret Information for Cas12a.
**Table S5.** Primer Information for Cas12a.

## Data Availability

All supporting data from this study are available in the article and Supplementary Information files. The data and scripts used are saved in GitHub https://github.com/ymx0723/Cas12fam.git. Supplementary materials (methods, figures, tables, graphical abstract, slides, videos, Chinese translated version and update materials) may be found in the online DOI or iMeta Science http://www.imeta.science/imetaomics/.
