# Supplementary material for: Metagenomic mining reveals novel Cas12 subtypes and their evolutionary diversification
Source: IMetaOmics. 2025 Oct 7;3(1):e70057. doi: 10.1002/imo2.70057 (PMC13674389; doi:10.1002/imo2.70057)
Supplement: Supplementary file 1 [file IMO2-3-e70057-s002.docx]

Supporting information to

**Metagenomic mining reveals** **novel Cas12 subtypes and their evolutionary diversification**

**Running title**: Novel Cas12 subtypes and their evolutionary diversification

Meixia Yu^1#^, Zilong Zhao ^1#^, Peiyang Li^1^, Yunpeng Teng^1^, Feng Xu^1^, Zhimeng Li^1^, Zhijia Yang^2^, Hainan Zhao^2^, Huanbin Zhou^3,4,5^, Lanqin Xia^4,6^, Jinsheng Lai^1,2,7,8,9^, Beibei Xin^1,9*^

^1^State Key Laboratory of Maize Bio-breeding, National Maize Improvement Center, Department of Plant Genetics and Breeding, China Agricultural University, Beijing 100193, China

^2^Frontiers Science Center for Molecular Design Breeding (Ministry of Education), China Agricultural University, Beijing 100193, China

^3^State Key Laboratory for Biology of Plant Diseases and Insect Pests, Institute of Plant Protection, Chinese Academy of Agricultural Sciences, Beijing 100193, China

^4^Ministry of Agriculture and Rural Affairs Key Laboratory of Gene Editing Technologies (Hainan), National Nanfan Research Institute, Chinese Academy of Agricultural Sciences, Sanya 572024, China

^5^Scientific Observing and Experimental Station of Crop Pests in Guilin, Ministry of Agriculture and Rural Affairs, Guilin 541399, China

^6^State Key Laboratory of Crop Gene Resources and Breeding, Institute of Crop Sciences (ICS), Chinese Academy of Agricultural Sciences (CAAS), Beijing 100081, China

^7^Center for Crop Functional Genomics and Molecular Breeding, China Agricultural University, Beijing 100193, China

^8^Sanya Institute of China Agricultural University, Sanya 572025, China

^9^Ministry of Agriculture and Rural Affairs Key Laboratory of Gene Editing Technologies (Beijing), Beijing 100193, China

^#^These authors contributed equally to this work: Meixia Yu, Zilong Zhao

^*^Correspondence: bxin@cau.edu.cn (Beibei Xin)

**M****ETHODS**

**Identification of novel Cas12 proteins**

8 TB of metagenomic data from the Integrated Microbial Genomes with Microbiome Samples (IMG/M: https://img.jgi.doe.gov/m/) [1,2] and assembled bacterial and archaeal genomes from the NCBI GenBank (https://www.ncbi.nlm.nih.gov/genbank/) [3] were collected. Contigs greater than 3 kilobases were chosen to ensure comprehensive genomic context for CRISPR array identification and subsequent analyses. CRISPR arrays within the metagenomic sequences were annotated using Piler-CR (V1.06) [4] and MinCED (V0.4.2) [5] with default settings. Proteins located within 10 kilobases upstream or downstream of each CRISPR locus were predicted using Prodigal (V2.6.1) [6]. Proteins shorter than 200 amino acids were excluded to filter out those with low functional potential. The remaining CRISPR-associated proteins were annotated using HMMER (V3.3.1) [7], with an e-value threshold set to 1 × 10^-2^ to identify potential Cas12 proteins against the Pfam profiles present in the NCBI’s Conserved Domain Database (<https://www.ncbi.nlm.nih.gov/Structure/cdd/cdd.shtml>) [8]. This step aimed to identify candidate Cas12 proteins that contained the type V nuclease RuvC domain.

A PSI-BLAST (V2.15.0+) [9] search was performed with an e-value threshold of 1 × 10^-7^ to identify new members of published type V within the candidate Cas12 proteins pool. Given the extensive computational demands associated with constructing phylogenetic trees from large datasets, MMseqs2 (V15.6f452) [10] was applied to streamline the process. By clustering proteins with a sequence similarity threshold of 0.5, we effectively reduced the dataset size to facilitate more manageable subsequent analyses. IQ-TREE2 (V2.3.6) [11] was used to construct the phylogenetic tree, aiming to explore of evolutionary relationships among the Cas12 subtypes.

To minimize false positives from highly similar TnpB proteins, we downloaded TnpB proteins from Altae-Tran et al. [12] and excluded sequences with greater than 80% similarity, which is comparable to the similarity between Cas14 and TnpB reported in Jennifer Doudna’s study [2]. In addition, all candidate proteins located adjacent to functional genes such as IS200/605 family Y1 TnpA, serine recombinases, DDE integrases, or tyrosine integrases were excluded to further eliminate canonical TnpB proteins and improve the specificity of Cas12 identification.

The protein sequences of 32 Cas12 subtypes and 4 Cas9 subtypes were analyzed with EMBOSS (V6.6.0.0) with a gap-open penalty of 10 and a gap-extension penalty of 0.5 [13]. The pairwise sequence similarities were categorized into four intervals based on percentage identity: 0−25%, 25−50%, 50−75%, and 75−100%. The proportion of proteins within each similarity range was calculated and presented.

**Characterization of Cas12 proteins, CRISPR repeat motifs, and tracrRNA dependence**

Protein and CRISPR array sequences from the diverse Cas12 subtypes were collected and subjected to multiple sequence alignments using the MAFFT (V7.526) program with the L-INS-i algorithm [14].To visualize the repeat patterns of CRISPR arrays, sequence logos were generated using WebLogo (V3.7.12) [15], which provides graphical representations of the alignment’s conserved regions. Conserved motifs were identified based on a threshold occurrence probability of 80%. Their functional relevance and conservation were validated through extensive literature review and comparative analysis with known motifs from the Pfam database. Cas12 protein sequences were analyzed using the ESM‑2 protein language model to assess tracrRNA dependence, following the approach described by Li et al. [16].

**Cas12fam: protein domain annotation of Cas12 proteins**

Cas12fam begins by collecting all available PDB structures of Cas12 proteins (Table S3) and preparing two structural databases: one containing full-length Cas12 protein structures and another comprising 18 domain structures (domains D= {WED1, WED2, WED3, REC1, REC2, REC3, PI, RuvC1, RuvC2, RuvC3, BH, NUC1, NUC2, PRS, STP, HNC, TSL, LC}) derived from these proteins. To annotate the domain architecture of a newly identified Cas12 protein, its structure is first predicted using AlphaFold2 [17]. Only structures with pLDDT scores above 70 were kept for downstream analyses. The predicted structure is then aligned against the full-length Cas12 protein database using DALI [18] and against the 18-domain database using Foldseek [19]. These alignments generate amino acid scoring matrices $M_{ij}^{1}$ and $M_{ij}^{2}$ respectively, where $i (1 \leq i \leq N)$ represents the position of an amino acid in a protein of length $N$ and $j (j \in D$) corresponds to one of the 18 domains. By adding $M_{ij}^{1}$ and $M_{ij}^{2}$, a comprehensive protein amino acid scoring matrix $M_{ij}$ was obtained, integrating the results of both global and local alignments. A higher score indicates a stronger match with a specific target structural domain.

Sword2 [20] was first employed for primary partitioning of domain intervals, for example, a protein might be partitioned into $K$ domain intervals. Sword2 utilizes two main rules based on the contact probability map to define structural domain boundaries: 1) strong contacts between amino acids within a domain; 2) minimal contacts between domains. For each structural domain interval $k (1 \leq k \leq K)$, as defined by Sword2, we identify the domain $j (j \in D)$ with the highest score for each amino acid within the interval. The domain type of interval $k$ is then determined by the most frequently occurring domain $j$within that interval. This process can be expressed mathematically as follows:

$j_{k}=\underset{j\in D}{\mathrm{argmax}} (\sum_{i in interval k} \delta_{ij})$,

where$\delta_{ij}$ is an indicator function that equals 1 if the domain $j$ has the highest score for amino acid $i$, and 0 otherwise. $j_{k}$represents the domain type assigned to interval $k$. Considering that Sword2 may not provide accurate boundaries for domains frequently in close spatial proximity, such as the RuvC, WED1, and NUC, a sliding window approach was employed to further refine these domain boundaries. For RuvC and NUC domains, for instance, windows of 20 amino acids were analyzed, and the domain type $j$ was assigned based on the most frequently occurring domain within each window. After annotating all windows in the RuvC domain, adjacent intervals with the same domain type were merged, resulting in more precise domain boundaries. It should be noted that the window size varies depending on the domain type, with 5 amino acids for the WED1 domain. The refined structural domain annotations were integrated with the initial domain annotations from Sword2, producing a comprehensive structural domain annotation for Cas12 proteins. The implementation of Cas12fam was deposited at Github repository at https://github.com/ymx0723/Cas12fam.git.

The accuracy of Cas12fam was evaluated using a leave-one-out cross-validation strategy. In each iteration, one protein was excluded from both the full-length structure in the global database and the corresponding single-domain structure in the local database. This excluded protein served as the test sample. Cas12fam was then applied to the remaining proteins, and the predicted domain annotations for the test sample were compared with the experimentally determined domains. This process was repeated iteratively for all 21 proteins in the dataset.

To evaluate the accuracy of Cas12fam’s domain annotations, the overlap between the predicted and true domains was quantified. The overlap was calculated by measuring the intersection of the predicted domain boundaries with those of the true domains. An annotation was deemed successful if the overlap between the predicted and true domain boundaries fell within 70% to 130% of the true domain length.

**Phylogenetic analysis**

To investigate the phylogenetic relationships among the 32 Cas12 subtypes, we utilized previously established alignments. Cluster-to-cluster similarity scores were calculated using HHsearch (V3.3.0) [7]. These similarity scores were normalized by the minimum self-scores of the clusters to ensure accuracy. The distance between subtypes was calculated through the formula 𝑑 = −ln 𝑠. This transformation allows for the use of these distances in constructing an unweighted pair-group method with arithmetic mean (UPGMA) dendrogram, which visually represents the phylogenetic relationships among the subtypes.

**Ka/Ks Calculation**

The integration of data prior to the calculation of Ka/Ks ratios was facilitated by ParaAT2.0 [21], which streamlined the preprocessing of genetic sequences. Protein sequences of gene pairs were aligned using MAFFT (V7.526) with the --auto algorithm [14]. Subsequently, Ka (nonsynonymous substitution rate) and Ks (synonymous substitution rate) values were calculated using KaKs_Calculator 2.0 [22], employing the Yang-Nielsen (YN) model.

**Habitats classification and saturation of Cas12 subtypes**

Metagenomic data were manually classified based on sampling locations downloaded from the Integrated Microbial Genomes with Microbiome Samples (IMG/M: https://img.jgi.doe.gov/m/). Sampling sites were grouped into 18 environments according to classification standards for environmental samples in the published literature and the sampling classification methods used in the JGI database (https://img.jgi.doe.gov/cgi-bin/m/main.cgi) [12,23−25]. To ensure data accuracy, we performed deduplication by removing protein sequences that originated from the same environment and shared identical sequences, thereby improving the precision of the analysis. As a result, each sampling environment contained only unique protein sequences.

To evaluate the saturation of Cas12 subtypes in each environment, we utilized a sample-based rarefaction curve analysis. Specifically, random sampling was performed from the deduplicated sequence set, with each iteration including 100 contigs. The cumulative number of Cas12 proteins was calculated at each step, and rarefaction curves was generated to visualize the saturation trend.

**Plasmid construction**

Human codon-optimized *LbCas12a* was synthesized and cloned to generate the *pCMV-LbCas12a* expression vector. The LbCas12a gene was inserted into the *BstX* I (NEB) and *BamH* I (NEB) restriction sites via homologous recombination. LbCas12a mutants (G834A and G991A) were generated using site-directed mutagenesis. To express the sgRNA, the *AAVS1*, *EMX1*, and *VEGFA* genes from the HeLa cell genome were selected as the targets (Table S4). Primers were designed to amplify the target fragment, which was subsequently inserted into the pU6-sgRNA vector at the *Bbs* I (NEB) site via homologous recombination (Table S5). The recombinant plasmids were purified using an endotoxin-free plasmid extraction kit (Omega) and prepared for further experiments.

**Cell culture, transfection**

HeLa cells were cultured in DMEM (Gibco) supplemented with 10% FBS (Gibco), 1% penicillin-streptomycin (Gibco), and 1% NEAA (Gibco). Cells were maintained at 37°C with 5% CO_2_. For plasmid transfection, HeLa cells were seeded in a 48-well plate at a density of 1.0 × 10^5^ cells per well. The next day, each well was transfected with 0.3 μg of protein plasmids, 0.2 μg of guide RNA plasmids, and 1 μL of Lipofectamine 3000 (Thermo Fisher). After 24 hours post-transfection, successfully transfected cells were selected by the addition of 1 μg/mL puromycin (InvivoGen) to the cell culture, and the cells were harvested for genomic extraction 48 hours later. The procedures for LbCas12a- and LbCas12a mutant-mediated human genome editing were carried out as described above.

**Indel efficiency analysis**

To precisely analyze the indel efficiency of these proteins, target genomic sequences were PCR-amplified using the 2× Hieff Canace® PCR Master Mix (YEASEN) with primer sets flanking the examined crRNA target sites, which contained an adaptor sequence. The first PCR products were subjected to a second round of PCR amplification with primers containing different barcode sequences. The NGS libraries were then pooled and purified by electrophoresis on a 1.5% agarose gel using a TIANgel Purification Kit (TIANGEN), with elution in 30 μL of ddH_2_O. These libraries were sequenced with 150-bp paired-end reads on a DNBSEQ-T7 instrument. Sequencing reads were analyzed using CRISPResso2 (V2.3.1) [26] with a quantification window centered at 1 bp and a plot window size of 50 bp.

**REFERENCES**

1. Chen, I. Min A., Ken Chu, Krishna Palaniappan, Manoj Pillay, Anna Ratner, Jinghua Huang, Marcel Huntemann, et al. 2019. “IMG/M v.5.0: an integrated data management and comparative analysis system for microbial genomes and microbiomes.” *Nucleic Acids Research* 47: D666-D677. <https://doi.org/10.1093/nar/gky901>

2. Harrington, Lucas B., David Burstein, Janice S. Chen, David Paez-Espino, Enbo Ma, Isaac P. Witte, Joshua C. Cofsky, Nikos C. Kyrpides, Jillian F. Banfield, Jennifer A. Doudna. 2018. “Programmed DNA destruction by miniature CRISPR-Cas14 enzymes.” *Science* 362: 839-842. <https://doi.org/10.1126/science.aav4294>

3. Benson, Dennis A., Mark Cavanaugh, Karen Clark, Ilene Karsch-Mizrachi, David J. Lipman, James Ostell, Eric W. Sayers. 2013. “GenBank.” *Nucleic Acids Research* 41: D36-D42. <https://doi.org/10.1093/nar/gks1195>

4. Edgar, Robert C. 2007. “PILER-CR: fast and accurate identification of CRISPR repeats.” *BMC Bioinformatics* 8: 18. <https://doi.org/10.1186/1471-2105-8-18>

5. Bland, Charles, Teresa L. Ramsey, Fareedah Sabree, Micheal Lowe, Kyndall Brown, Nikos C. Kyrpides, Philip Hugenholtz. 2007. “CRISPR Recognition Tool (CRT): a tool for automatic detection of clustered regularly interspaced palindromic repeats.” *BMC Bioinformatics* 8: 209. <https://doi.org/10.1186/1471-2105-8-209>

6. Hyatt, Doug, Gwo-Liang Chen, Philip F. LoCascio, Miriam L. Land, Frank W. Larimer, Loren J. Hauser. 2010. “Prodigal: prokaryotic gene recognition and translation initiation site identification.” *BMC Bioinformatics* 11: 119. <https://doi.org/10.1186/1471-2105-11-119>

7. Potter, Simon C., Aurélien Luciani, Sean R. Eddy, Youngmi Park, Rodrigo Lopez, Robert D. Finn. 2018. “HMMER web server: 2018 update.” *Nucleic Acids Research* 46: W200-W204. <https://doi.org/10.1093/nar/gky448>

8. Mistry, Jaina, Sara Chuguransky, Lowri Williams, Matloob Qureshi, Gustavo A Salazar, Erik L. L. Sonnhammer, Silvio C. E. Tosatto, et al. 2021. “Pfam: the protein families database in 2021.” *Nucleic Acids Research* 49: D412-D419. <https://doi.org/10.1093/nar/gkaa913>

9. Altschul, Stephen F., Thomas L. Madden, Alejandro A. Schäffer, Jinghui Zhang, Zheng Zhang, Webb Miller, David J. Lipman. 1997. “Gapped BLAST and PSI-BLAST: a new generation of protein database search programs.” *Nucleic Acids Research* 25: 3389-3402. <https://doi.org/10.1093/nar/25.17.3389>

10. Steinegger, Martin, Johannes Söding. 2017. “MMseqs2 enables sensitive protein sequence searching for the analysis of massive data sets.” *Nature Biotechnology* 35: 1026-1028. <https://doi.org/10.1038/nbt.3988>

11. Minh, Bui Quang, Heiko A. Schmidt, Olga Chernomor, Dominik Schrempf, Michael D. Woodhams, Arndt von Haeseler, Robert Lanfear. 2020. “IQ-TREE 2: new models and efficient methods for phylogenetic inference in the genomic era.” *Molecular Biology and Evolution* 37: 1530-1534. <https://doi.org/10.1093/molbev/msaa015>

12. Altae-Tran, Han, Sergey A. Shmakov, Kira S. Makarova, Yuri I. Wolf, Soumya Kannan, Feng Zhang, Eugene V. Koonin. 2023. “Diversity, evolution, and classification of the RNA-guided nucleases TnpB and Cas12.” *Proceedings of the National Academy of Sciences* 120: e2308224120. [https://doi.org/10.1073/pnas.2308224120](https://doi.org/doi:10.1073/pnas.2308224120)

13. Rice, Peter, Ian Longden, Alan Bleasby. 2000. “EMBOSS: The European molecular biology open software suite.” *Trends in Genetics* 16: 276-277. <https://doi.org/10.1016/S0168-9525(00)02024-2>

14. Katoh, Kazutaka, Kazuharu Misawa, Kei‐ichi Kuma, Takashi Miyata. 2002. “MAFFT: a novel method for rapid multiple sequence alignment based on fast Fourier transform.” *Nucleic Acids Research* 30: 3059-3066. <https://doi.org/10.1093/nar/gkf436>

15. Crooks, Gavin E., Gary Hon, John-Marc Chandonia, Steven E. Brenner. 2004. “WebLogo: a sequence logo generator.” *Genome Research* 14: 1188-1190. <https://doi.org/10.1101/gr.849004>

16. Li, Wenhui, Xianyue Jiang, Wuke Wang, Liya Hou, Runze Cai, Yongqian Li, Qiuxi Gu, et al. 2024. “Discovering CRISPR-Cas system with self-processing pre-crRNA capability by foundation models.” *Nature Communications* 15: 10024. <https://doi.org/10.1038/s41467-024-54365-0>

17. Jumper, John, Richard Evans, Alexander Pritzel, Tim Green, Michael Figurnov, Olaf Ronneberger, Kathryn Tunyasuvunakool, et al. 2021. “Highly accurate protein structure prediction with AlphaFold.” *Nature* 596: 583-589. <https://doi.org/10.1038/s41586-021-03819-2>

18. Holm, Liisa. 2022. “Dali server: structural unification of protein families.” *Nucleic Acids Research* 50: W210-W215. <https://doi.org/10.1093/nar/gkac387>

19. van Kempen, Michel, Stephanie S. Kim, Charlotte Tumescheit, Milot Mirdita, Jeongjae Lee, Cameron L. M. Gilchrist, Johannes Söding, Martin Steinegger. 2024. “Fast and accurate protein structure search with Foldseek.” *Nature Biotechnology* 42: 243-246. <https://doi.org/10.1038/s41587-023-01773-0>

20. Cretin, Gabriel, Tatiana Galochkina, Yann Vander Meersche, Alexandre G de Brevern, Guillaume Postic, Jean-Christophe Gelly. 2022. “SWORD2: hierarchical analysis of protein 3D structures.” *Nucleic Acids Research* 50: W732-W738. <https://doi.org/10.1093/nar/gkac370>

21. Zhang, Zhang, Jingfa Xiao, Jiayan Wu, Haiyan Zhang, Guiming Liu, Xumin Wang, Lin Dai. 2012. “ParaAT: a parallel tool for constructing multiple protein-coding DNA alignments.” *Biochemical and Biophysical Research Communications* 419: 779-781. <https://doi.org/10.1016/j.bbrc.2012.02.101>

22. Wang, Dapeng, Yubin Zhang, Zhang Zhang, Jiang Zhu, Jun Yu. 2010. “KaKs_Calculator 2.0: a toolkit incorporating gamma-series methods and sliding window strategies.” *Genomics, Proteomics & Bioinformatics* 8: 77-80. <https://doi.org/10.1016/S1672-0229(10)60008-3>

23. Santos-Júnior, Célio Dias, Marcelo D. T. Torres, Yiqian Duan, Álvaro Rodríguez del Río, Thomas S. B. Schmidt, Hui Chong, Anthony Fullam, et al. 2024. “Discovery of antimicrobial peptides in the global microbiome with machine learning.” *Cell* 187: 3761-3778.e3716. <https://doi.org/10.1016/j.cell.2024.05.013>

24. Li, Yisong, Yeshun Fan, Xiaofang Ma, Ying Wang, Jie Liu. 2023. “Metagenomic survey reveals global distribution and evolution of microbial sialic acid catabolism.” *Frontiers in Microbiology* 14: 1267152. <https://doi.org/10.3389/fmicb.2023.1267152>

25. Aßhauer, Kathrin P., Heiner Klingenberg, Thomas Lingner, Peter Meinicke. 2014. “Exploring neighborhoods in the metagenome universe.” *International Journal of Molecular Sciences* 15: 12364-12378. <https://www.mdpi.com/1422-0067/15/7/12364>

26. Clement, Kendell, Holly Rees, Matthew C. Canver, Jason M. Gehrke, Rick Farouni, Jonathan Y. Hsu, Mitchel A. Cole, et al. 2019. “CRISPResso2 provides accurate and rapid genome editing sequence analysis.” *Nature Biotechnology* 37: 224-226. <https://doi.org/10.1038/s41587-019-0032-3>

**Figure S1 Identification and characterization of Cas12 effectors from metagenomic data.** (A) Workflow of the computational pipeline for identifying Cas12 effectors from metagenomic datasets. (B) A circular plot illustrating the sequence identity relationships among 32 Cas12 subtypes, with four Cas9 subtypes (Type II) included for comparison. Colors indicate identity ranges (0–25%, 25–50%, 50–75%, 75–100%) within and between subtypes. (C) Length variation across Cas12 subtypes. The graph shows the full protein length (green) and C-terminal lobe length (yellow) for each Cas12 subtype. Error bars represent the interquartile range (IQR) for sequences within each subtype. (D) Box plots showing the variation in GC content (red) and repeat length (blue) among different Cas12 subtypes. Each box represents the interquartile range (IQR), with whiskers extending to the farthest data point that is not considered an outlier (based on the 1.5 × IQR rule), and individual outliers are shown as dots. The figure illustrates significant diversity in GC content and repeat length across Cas12 subtypes, suggesting structural and functional variability within the CRISPR-Cas system. (E) t-Distributed Stochastic Neighbor Embedding (t-SNE) plot of 32 Cas12 subtypes based on sequence similarity. Each dot represents a Cas12 protein, colored according to its subtype. (F) t-SNE plot of 32 Cas12 subtypes based on structural similarity. The dots are colored by subtype, highlighting the relationships and clustering of Cas12 proteins according to structural features.

**Figure S2 Sequence logos of direct repeat in Cas12 subtypes.** This figure displays sequence logos representing the direct repeats (DR) across various Cas12 subtypes. For each subtype, the nucleotide frequency at each position is illustrated, with taller letters indicating higher sequence conservation.

**Figure S3 Structural overview and comparative analysis of the REC lobe across Cas12 subtypes.** This figure shows the structures of various Cas12 subtypes, where published structures are used for subtypes with available data, and AlphaFold2 predictions are applied for subtypes lacking published structures. The structures are color-coded based on the domains of the REC lobe. The WED domain is represented in yellow, other domains are shown in gray.

**Figure S4** **Functional motifs identified in Cas12 subtypes.** The diagram displays known functional motifs (Cas12C1−Cas12C15) collected from previously published studies, represented by various colors along the sequence. Predicted functional motifs for subtypes Cas12C16−Cas12C32 were inferred based on the identified motifs from Cas12C1−Cas12C15.

**Figure S5** **Distribution, diversity, and selection pressure analysis of Cas12 proteins**. (A) Distribution of Cas12 proteins compared to overall metagenomic proteins across 18 sampled environments. The proportion of Cas12 proteins (pink bars) is contrasted with the metagenomic background (blue bars) for each environment type, with statistically significant enrichment indicated by asterisks (**p* < 0.05). The statistical test used is the Chi-square test, which is a two-tailed test, and p-values below 0.05 indicate significant differences in the distribution of Cas12 proteins compared to the metagenomic background. (B) Rarefaction curves indicating the discovery rate of Cas12 proteins across different environments as a function of sample size (100 random contigs per iteration). The inset magnifies low-richness environments. (C) Box plots of Ka/Ks ratios for each Cas12 subtype. Boxes show the interquartile range with the median indicated by the central line; whiskers extend to the farthest data point that is not considered an outlier (based on a 1.5 × IQR rule). The red dashed line marks Ka/Ks = 1. Ratios greater than 1 suggest positive selection, whereas ratios below 1 indicate purifying selection.
